# Cerebrotendinous xanthomatosis with tremor as the main manifestation: A case report

**DOI:** 10.1097/MD.0000000000037976

**Published:** 2024-04-26

**Authors:** Wei Zhao, Jie Han, Dingbo Tao, Hongliang Zheng

**Affiliations:** a Neurology Department, Ji AO Brain Hospital of Siping, Si Ping, Jilin Province, China; b Neurological Intensive Care Unit Department, The First Affiliated Hospital of Dalian Medical University, Da Lian, Liaoning Province, China.

**Keywords:** case report, cataract, cerebrotendinous xanthomatosis, epilepsy, tendon xanthoma, tremor

## Abstract

**Introduction:**

Cerebrotendinous xanthomatosis (CTX) is an autosomal recessive lipid metabolism disorder. It is caused by a defect in the sterol-27-hydroxylase gene, leading to the deposition of cholesteryl and bile alcohol in large amounts, causing a variety of clinical manifestations; however, tremor as the main manifestation of CTX has not been reported.

**Patient’s concerns and clinical findings:**

Herein, we report a 27-year-old woman, who developed head and body tremors at the age of 12 years. Many hospitals misdiagnosed her condition as idiopathic tremor and Parkinson disease, with a poor curative effect.

**Primary diagnosis and intervention:**

We diagnosed her with CTX and treated with chenodeoxycholic acid and clonazepam.

**Conclusion:**

The patient’s condition considerably improved. This case could help avoid misdiagnosis and mistreatment in clinical practice.

## 1. Introduction

Cerebrotendinous xanthomatosis (CTX) is an autosomal recessive lipid metabolism disorder caused by a defect in the sterol-27-hydroxylase gene (*CYP27A1*) on chromosome 2. Mitochondrial cholesterol 27-hydroxylase, encoded by this gene, catalyzes the synthesis of bile acids from cholesterol in various tissues and cells, other than the liver. A mutation in this gene can reduce the activity of cholesterol 27-hydroxylase, resulting in the deposition of bile acid precursors (cholesterol and bile alcohol) in soft tissues and causing extensive damage to the nervous system and other systems. Such damages lead to adolescent cataracts, xanthomatosis of the tendon, early-onset atherosclerosis, mental retardation, and cerebellar dysfunction.^[1–3]^ CTX is rare, with <600 cases reported globally,^[4]^ and the main manifestation of head and body tremors is even rarer and has not been reported. Herein, we report a patient with CTX who was admitted to our hospital. Our findings could help guide the clinical diagnosis and treatment of this disease. The study was approved by the Ethics Committee of Ji AO Brain Hospital of Siping and was performed in accordance with the recommendations of the Declaration of Helsinki. Written informed consent was obtained from the patient and the guardian.

## 2. Case report

A 27-year-old woman was admitted to our hospital for paroxysmal limb convulsions, mental decline for 20 years, and tremors for 15 years. The patient first experienced limb convulsions 20 years ago (March 2003), and the symptoms included limb rigidity, foaming at the mouth, and rearward head movement, which lasted for approximately 3 to 5 minutes before relief. Subsequently, the patient experienced a slight mental decline, poor learning ability, poor test results, occasional inability to communicate, inability to understand her work, and a lack of eccentricity. Her condition remained the same in March 2004, and in March 2008, a relapse occurred, with limb rigidity, foaming at the mouth, and double-eyed gaze, which lasted 3–5 min. When the attacks became more frequent, 3 to 4 times a month, the family began seeking medical treatment. In September 2008, head and body tremors began to gradually present, and her voice grew more tense with more obvious voice trembling, although this did not affect her daily activities. Twelve years ago, her binocular vision gradually declined, and the patient was diagnosed with binocular cataracts in a hospital. During the course of the disease, the patient did not present movement retardation, posture gait abnormality, walking instability, headache, neck pain and chest pain, chest tightness, shortness of breath, or difficulty in breathing. Although the patient’s family consulted the doctor at that hospital many times, a clear diagnosis could not be made. She was then transferred to our hospital with a classification of “pyramidal system disease.”

**Figure 1.**
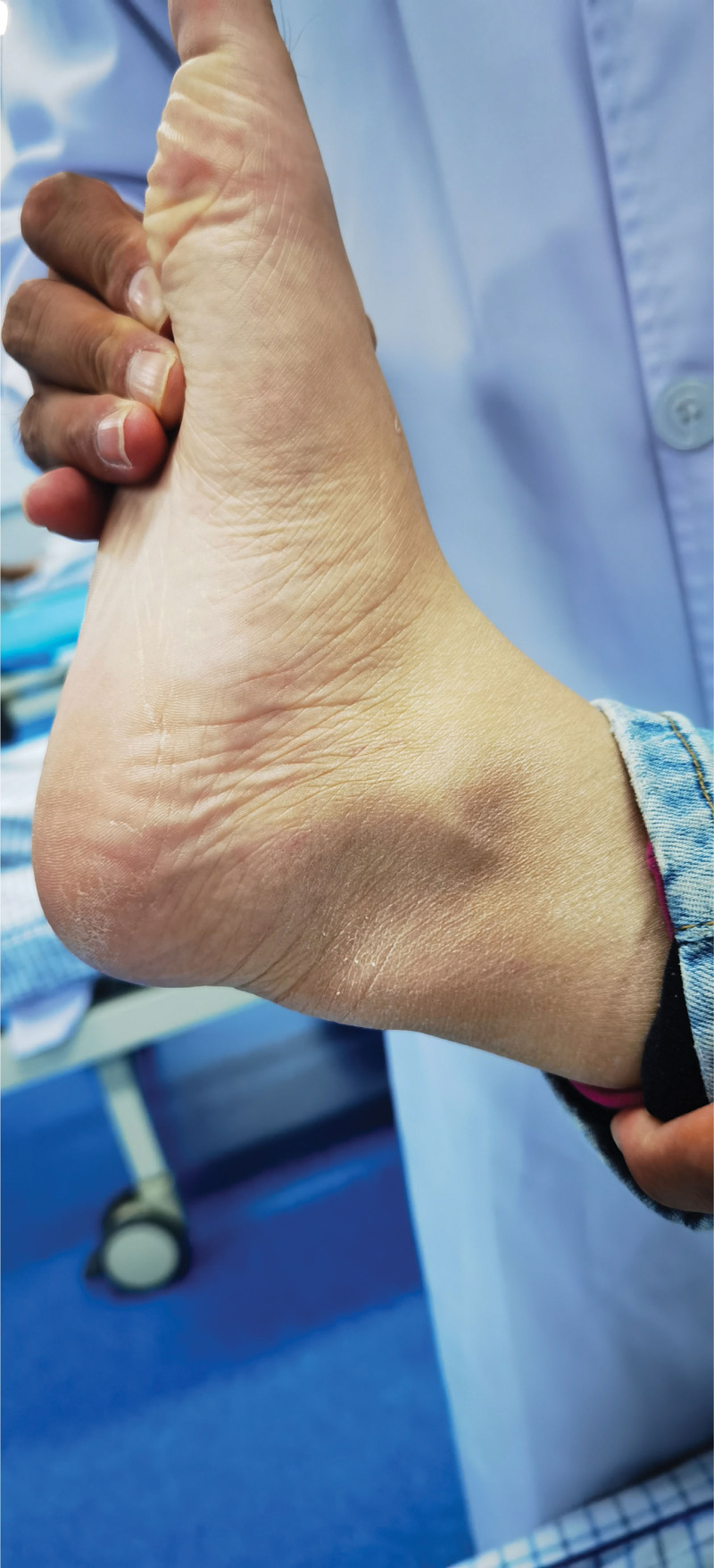
Achilles tendon mass in the patient.

**Figure 2.**
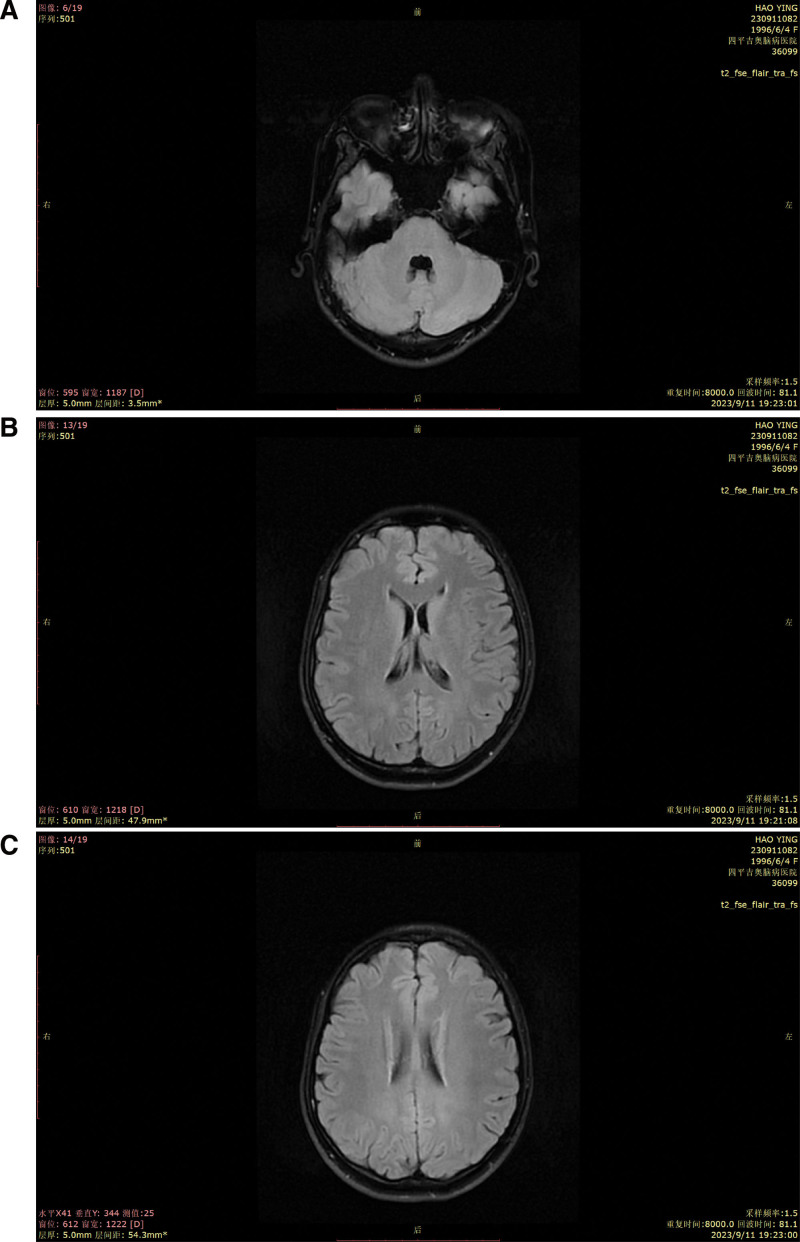
Brain MR exam. T2-weighted MRI showed (A) no abnormalities in the cerebellum and (B and C) white matter lesions around the posterior horn of the lateral ventricle on both sides. MRI = magnetic resonance imaging.

**Figure 3.**
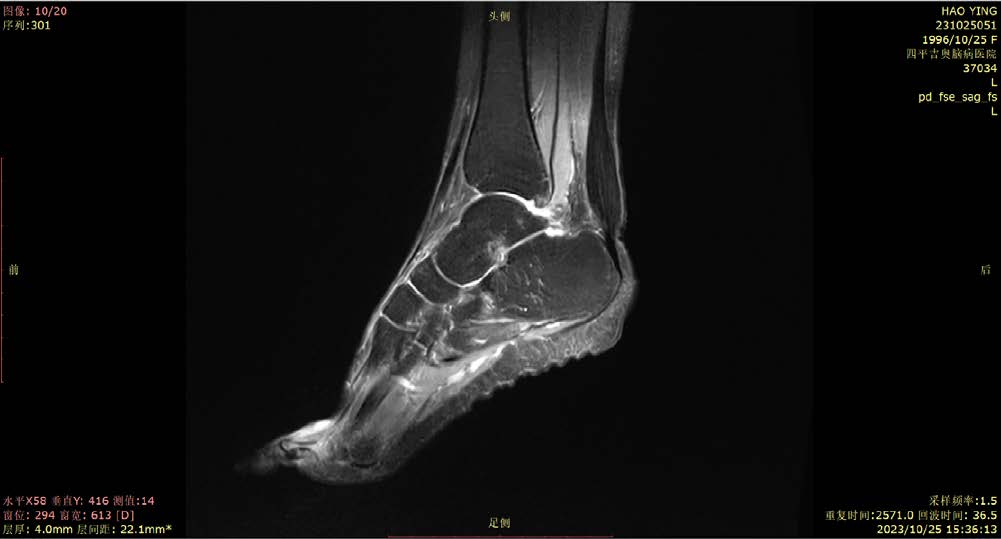
MRI proton weighted sequence of the patient’s Achilles tendon presenting an Achilles tendon mass. MRI, magnetic resonance imaging. MRI = magnetic resonance imaging.

**Figure 4.**
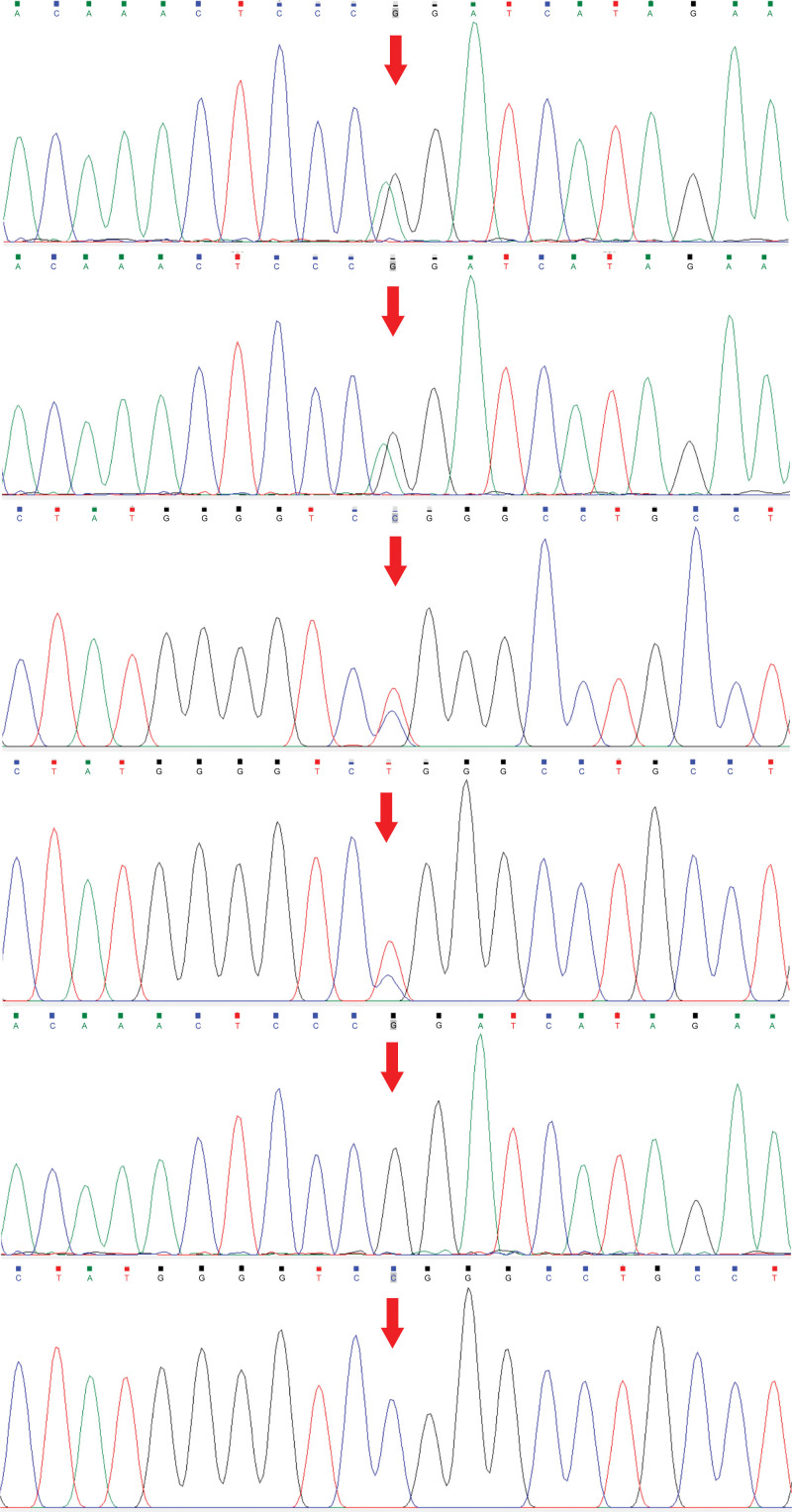
*CYP27A1* genetic test results of the patient’s family. (A) c. 1214G>A heterozygous mutation in the proband. (B) c. 1214G>A heterozygous mutation in the proband’s father. (C) c. 1420C>T heterozygous mutation in the proband. (D) c. 1420C>T heterozygous mutation in the proband’s mother. (E) No mutation at the c. 1214 site was observed in the proband’s sister. (F) No mutation at the c. 1420 site was observed in the proband’s other sister.

Her 10-year medical history was as follows: gradual depression, lack of happiness, tension, worry, fear, irritability, sleep disorders, and other symptoms, with no formal diagnosis or treatment. The patient did not have hypertension, coronary heart disease, diabetes, or cerebral infarction, nor a history of surgery, trauma, blood transfusion, hepatitis, tuberculosis, or other infectious diseases. The patient had no relevant family medical history. Her physical examination findings were as follows: temperature, 36.2 °C; heart rate, 88 times/min; breath, 18 times/min; blood pressure, 120/70 mm Hg, binocular uncorrected visual acuity, 0.08; emaciation; bilateral Achilles tendon solid mass (as shown in Fig. 1); and cardiopulmonary abdominal examination without abnormalities. She presented clear consciousness, fluent speech, cognitive decline, limited computing power, and memory and executive function decline. Her cranial nerve examinations showed no obvious abnormalities. Her limb muscle strength was of grade 5, and she had a normal muscle volume, slightly low muscle tension, body tremors, and bilateral tendon reflex symmetrical extraction. Her right finger nose test, heel–knee test instability, and left side findings were normal. No deep or superficial sensory disorders were observed. She presented a bilateral Chaddock sign, and was Babinski sign negative and bilateral ankle cramp positive. Her eyes presented the Romberg sign when opened and were stable when closed, and she could walk in a straight line steadily. Her auxiliary examination revealed the following blood lipid levels: cholesterol, 5.28 mmol/L; low-density lipoprotein, 3.5 mmol/L; and apolipoprotein, B1.19 g/L. Her liver function signs were as follows: total bilirubin, 23.7 µmol/L; total bile acid, 15 µmol/L; and glycocholic acid, 4.24 µg/mL. Her routine blood findings were as follows: red blood cell count, 3.34 × 10^12^/L; hemoglobin, 114 g/L; hematocrit, 34.6%; average red blood cell volume, 103.5 fL; average red blood cell hemoglobin content, 34.1 pg; and potassium ion concentration, 3.15 mmol/L. No obvious abnormalities were observed in her renal function, thyroid function, and the 9 items related to antinuclear antibody levels. The Wechsler Adult Intelligence Scale scores were 55, 55, and 51. Her Hamilton Anxiety Scale score was 11 and depression scale score was 18. No electrocardiographic abnormalities were observed, and the electroencephalography findings revealed a small number of slow waves of 5 to 7 Hz. Her full abdominal color Doppler ultrasound showed a slightly rough liver parenchyma and multiple gallbladder polypoid lesions. Her head magnetic resonance imaging (MRI) findings are presented in Figure 2—bilateral periventricular leukoencephalopathy and a straight cervical curvature. Her Achilles tendon MRI revealed bilateral masses at the Achilles tendon (Fig. 3). The initial diagnosis was extrapyramidal disease and CTX. Detection of *CYP27A1* in the family (Fig. 4) revealed 2 heterozygous variants: c. 1214G>A (chr2-219679132, p.Arg405Gln) and c. 1420C>T (chr2-219679424, p.Arg474Trp). According to the latest version of the gene variation interpretation standards and guidelines published by the American College of Medical Genetics and Genomics, the variant was determined to be pathogenic. The father had a c. 1214G>A heterozygous variant, the mother had a c. 1420C>T heterozygous variant, and the patient’s sister had no variants. The patient was diagnosed with tendon xanthomatosis and was administered 250 mg tid oral goose deoxycholate, 2.5 mg bid oral clonazepam, 5 mg qd oral donepezil, and 20 mg Qn oral atorvastatin calcium, and was discharged. At her 2-month follow-up, the tremors had considerably improved, but her cognitive impairment had not improved.

## 3. Discussion

The prevalence of CTX differs in various populations: 1/36,072 to 1/75,601 in South Asia, 1/64,247 to 1/64,712 in East Asia, 1/71,677 to 1/148,914 in the United States, 1/134,970 to 1/461,358 in Europe, and 1/263,222 to 1/468,624 in Africa. As physicians have a poor understanding of the disease and often misdiagnose it, the incidence of the disease may be underestimated.^[5]^

According to the ClinVar database, mutations in *CYP27A1* occur in exons 2, 4, 6, 7, and 8; approximately 52.5% of the mutations occur in exons 6, 7, and 8, and the rest occur in exons 2 (23.9 %) and 4 (23.6%). Currently, 941 *CYP27A1* mutations have been found, and nearly 150 variants are considered pathogenic or possibly pathogenic. The HGMD database contains approximately 138 pathogenic or possibly pathogenic mutations of the gene. The *CYP27A1* mutations found in Chinese individuals are mainly concentrated in exon 2, and the most common mutations are c.410G>A (p.R137Q, 22.7%), c.379C>T (p.R127W, 18.2%), and c.1435C>T (p.R479C, 9%).^[3]^ It has also been reported that the c.1263 + 1G>A mutation is common in patients in China and may be a potential hotspot of CTX mutations in these patients.^[6]^ In Caucasians, mutations are often located in exons 4 to 8, with the most common being c.1183C>T and c.1213C>T.^[7]^ Although the gene mutations differ among races, the clinical features are similar. Sekijima et al^[8]^ found that gene mutations in Japanese patients were mainly concentrated in exons 7 and 8, with the most common mutations being c.1214G>A (p.R405Q, 31.6%), c.1421G>A (p.R474Q, 26.3%), and c.43 5G>T (p.G145G, 15.8%). c.1421G>A is associated with the classic type, c.1214G>A with the spinal cord type, and c.435G>T with the nonnervous CTX type. Among these mutations, c.1214G>A (p.R405Q) and c.435G>T (p.Gly145=) have also been identified in Chinese patients. Our patient had heterozygous mutations on chromosome 2. One of the mutation sites was c.1214G>A; however, no spinal cord-related symptoms were observed. The second mutation was c.1420C>T (p.Arg474Trp), and these 2 sites are uncommon in Chinese individuals. Notably, c.1214G>A^[9–12]^ and c.1420C>T^[13–15]^ have been previously reported.

In terms of clinical manifestations, 92% of patients mainly present neurological symptoms, including cognitive dysfunction, psychiatric symptoms, pyramidal tract signs (e.g., weakness), cerebellar signs (such as ataxia and dysarthria), and peripheral neuropathy. Furthermore, 60% of patients have bilateral cataracts, 68% have xanthelasma tendinae, 20% have chronic diarrhea, and 4% have osteoporosis.^[16]^ Epilepsy is an uncommon manifestation of CTX, but sometimes appears as an initial symptom; therefore, CTX should be considered as a possible cause of epileptic seizures. Parkinson’s syndrome is less common and usually appears at an older age.^[17]^ The clinical manifestations observed in our patient are rare among individuals with CTX, with epilepsy as the initial manifestation, and her main symptoms were head and body tremors, and there are no reports of these manifestations. Some studies have pointed out that epilepsy and Parkinson’s syndrome are the first neurological manifestations of CTX.^[18,19]^ However, the physical examination of this patient showed slightly low muscle tension, no motor retardation, and abnormal posture and gait, which are not consistent with Parkinson syndrome; therefore, tremor was classified as an independent symptom. Tremors are very rare in CTX, and there are only a few reports of patients presenting with bilateral upper limb tremors,^[20–24]^ without head and body tremors, suggesting that we should consider the possibility of CTX in patients with epilepsy and body tremors, especially in patients with cataract and xanthelasma tendinae. However, the pathogenesis of these tremors remains unclear. Rich et al^[21]^ reported a patient with mixed tremor of both upper limbs, which was considered to be related to cerebellar dentate nucleus lesions, and there was no abnormality in the cerebellum of this patient. Li et al^[24]^ reported a patient with obvious bilateral upper limb tremors, and head susceptibility weighted imaging (SWI) indicated bilateral iron deposition, which suggests that such tremors may relate to postsynaptic iron deposition; however, SWI was not performed in our patient.

Patient serum cholesterol and triglyceride levels are usually normal, whereas the level of cholesteryl is often significantly increased.^[25]^ Of the patients reported previously, 56.57% showed abnormal EEG findings, such as diffuse slow waves, which are common features in patients with CTX, and 76.72% of patients had abnormal head MRI findings.^[17]^ The main head MRI abnormalities include supratentorial and infratentorial atrophy, as well as a strong signal on the T2 FLAIR sequences in the subcortex, periventricular region, dentate cerebellar nucleus, and brainstem.^[26]^ Bilateral dentate nucleus and periventricular white matter hyperintensities are considered to be typical imaging features of CTX.^[27,28]^ In this patient, there were no abnormal blood lipid results. The EEG findings showed a small number of slow waves and the head MRI findings showed no obvious cortical atrophy and cerebellar lesions, in accordance with the findings reported previously. However, there were mild white matter lesions around the ventricle, and white matter pathology in patients with CTX may be caused by excessive cholesterol incorporation in the glial cell membrane and changes in myelin lipid composition.^[29,30]^ In addition, intracerebral lipid deposition related to xanthoma and local inflammatory reactions can damage myelinated axons, neuronal cell bodies, and neuronal integrity, and disrupt gray matter formation,^[31,32]^ resulting in neuronal loss and deterioration of cognitive function, which are related to white matter lesions.

At present, there is no recognized diagnostic standard for CTX, and physicians still need to make a preliminary diagnosis based on medical history, physical examination, main clinical manifestations, and related auxiliary examinations, and then confirm the diagnosis through gene sequencing. Chenodeoxycholic acid is the recognized standard treatment for this disease and can effectively reduce cholesterol levels, reverse biochemical abnormalities, and stabilize or improve disability progression and most neurological and non-neurological manifestations, and its long-term safety has been recognized.^[33–36]^ For the tremor symptoms in our patient, chenodeoxycholic acid and clonazepam showed good therapeutic effects. Notably, Rich et al^[21]^ reported a patient with Parkinson syndrome with bilateral upper limb tremors, in whom the tremor and Parkinson symptoms considerably improved after DBS implantation. If the tremor symptoms in a patient aggravate, these treatments should be considered.

In summary, the incidence of CTX is low, and its clinical manifestations cover a wide range. Owing to insufficient understanding, this condition can be easily misdiagnosed. This patient’s medical history was characterized by rare head and body tremors. In its early stages, CTX is more likely to be misdiagnosed as an extrapyramidal disease or another genetic disease. The initial symptoms of epilepsy, bilateral Achilles tendon xanthelasma, and cataract history suggest the disease, and a final genetic diagnosis should be made. Therefore, careful medical history taking and physical examination are very important, and the symptoms of a patient often improve after treatment with chenodeoxycholic acid and clonazepam. Here, we reported a case of xanthomatosis of a brain tendon with tremors as the prominent manifestation, and our findings will help clinicians avoid misdiagnosis and mistreatment and delay the progression of CTX.

## Acknowledgments

We would like to thank the patient for his participation. We would also like to thank Editage (www.editage.cn) for the English language editing.

## Author contributions

**Writing – original draft:** Wei Zhao.

**Writing – review & editing:** Jie Han, Dingbo Tao, Hongliang Zheng.
